# Non-occlusive intestinal ischemia in the ascending colon and rectum: a pediatric case occurring during encephalitis treatment

**DOI:** 10.1186/s40792-019-0592-y

**Published:** 2019-02-15

**Authors:** Noboru Oyachi, Takaki Emura, Fuminori Numano, Tomoko Tando, Tomohiro Saito, Yusuke Goto

**Affiliations:** 1 Department of Pediatric Surgery, Yamanashi Prefectural Central Hospital, 1-1-1 Fujimi, Kofu, Yamanashi 400-8506 Japan; 2 Department of Pediatrics, Yamanashi Prefectural Central Hospital, Kofu, Japan

**Keywords:** Non-occlusive mesenteric ischemia, NOMI, Pediatrics, Encephalitis, Colon lesion

## Abstract

**Background:**

Non-occlusive mesenteric ischemia (NOMI) is a rare and severe pathological condition that can cause intestinal necrosis without mechanical obstruction of the mesenteric artery. NOMI often develops during the treatment of severe disease in elderly patients and mostly occurs in the intestine supplied by the superior mesenteric artery (SMA). We experienced a 12-year-old patient with NOMI that was segmentally localized in the ascending colon and rectum during encephalitis treatment.

**Case presentation:**

A 12-year-old boy was hospitalized with limbic encephalitis. On day 41 after admission, he abruptly developed hypotension following diarrhea and fever, and presented abdominal distension. A computed tomography scan revealed pneumatosis intestinalis localized in the ascending colon and rectum coexisting with portal venous gas. The presence of peritoneal signs required an emergency laparotomy. Intraoperatively, skip ischemic lesions were found in the ascending colon and the rectum without bowel perforation. SMA and superior rectal arterial pulsation were present, and the patient was diagnosed with NOMI. The remaining colon, from the transverse to the sigmoid colon, appeared intact. We performed a distal ileostomy without bowel resection. Postoperative colonoscopies were carried out and revealed rectal and ascending colon stenosis with ulceration but demonstrated the patency of the two lesions. We confirmed the improvement of the transient bowel strictures; therefore, the ileal stoma was closed 14 months after the previous laparotomy.

**Conclusion:**

NOMI can be present in childhood during encephalitis treatment and can be segmentally localized in the ascending colon and the rectum. Although NOMI is most often seen in elderly patients, we should also consider the possibility of NOMI when pediatric patients with severe illness manifest abdominal symptoms.

## Background

Non-occlusive mesenteric ischemia (NOMI) can lead to intestinal necrosis without mechanical obstruction of the mesenteric artery [1]. NOMI is a life-threatening disorder with a high mortality rate of approximately 50% [2, 3]. It is predominant in elderly patients with severe disease and of poor general condition. NOMI frequently presents in the small intestine and the right-side colon, where the mesenteric blood flow is supplied by the superior mesenteric artery (SMA) [4].

To date, there have been few reports of NOMI in pediatric patients [5], and there are only a few case reports of NOMI localized in the colon [6, 7]. We report here a pediatric case of NOMI that manifested skip lesions in the ascending colon and rectum and was treated by salvage surgery during the treatment of limbic encephalitis.

## Case presentation

A 12-year-old boy was hospitalized with complaints of a headache and high fever accompanied by psychosis, delirium, and indistinct consciousness. He was diagnosed with limbic encephalitis, which is an autoimmune disorder characterized by inflammation of the limbic area in the brain. His symptoms became exacerbated, and he required intensive therapies including high-dose steroid and catecholamine administration.

**Fig. 1:**
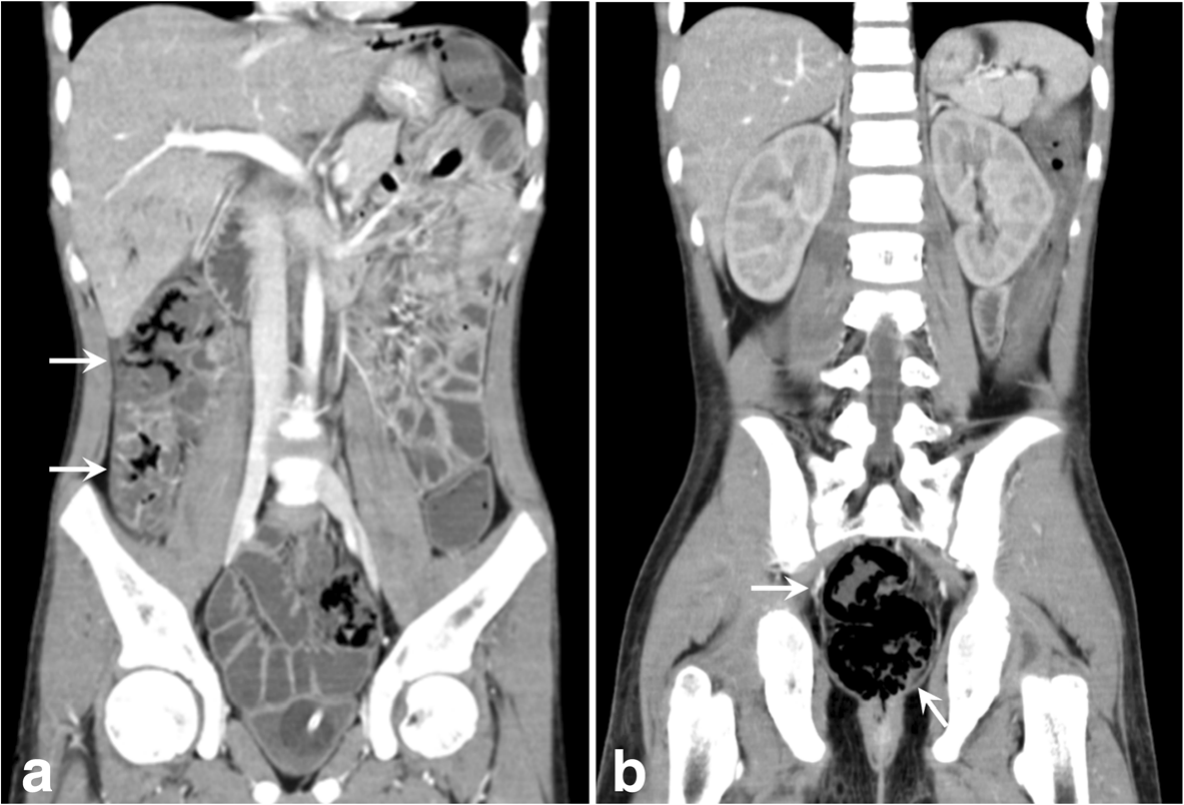
Contrast-enhanced abdominal computed tomography (CT). Emergency CT scans revealed pneumatosis intestinalis (arrows) localized in the ascending colon (**a**) and the rectum (**b**) coexisting with portal venous gas

Despite the continuous therapeutic support mentioned above, he abruptly developed hypotension following diarrhea, fever, and abdominal distension on day 41 after admission. Metabolic acidosis (pH 7.34, base excess − 7.0 mmol/L) was confirmed by blood gas analysis, and highly elevated CPK 11800 U/L, AST 461 U/L, ALT 201 U/L, and LDH 1034 U/L values were revealed by a blood chemistry profile. An emergency CT scan revealed pneumatosis intestinalis localized in the ascending colon and rectum coexisting with portal venous gas (Fig. 1). While the root of the SMA and the inferior mesenteric arterial (IMA) flow was maintained, the peripheral blood flow was attenuated adjacent to the non-contrast-enhanced ascending colon and rectum.

**Fig. 2:**
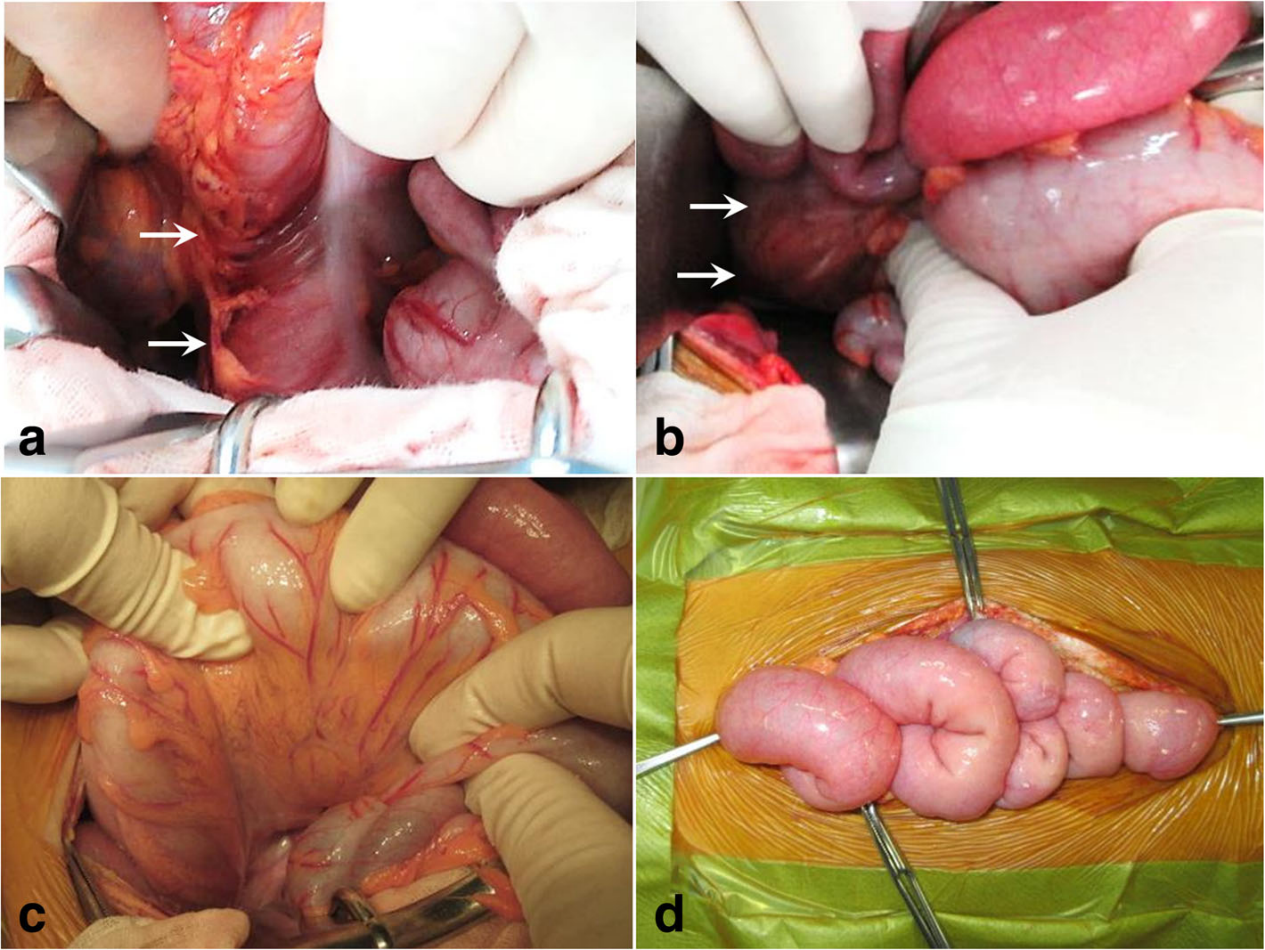
Operative findings. There were skip ischemic lesions (arrow) in the ascending colon (**a**) and the rectum (**b**) without bowel perforation. The remaining colon, from the transverse to the sigmoid colon, appeared intact (**c**). Bowel color and peristalsis were restored in the small bowel (**d**)

Although intraabdominal free air was not detected in the CT scan, the massive ascites and progressing peritoneal signs with muscular guarding required an emergency laparotomy for suspected mesenteric ischemia and bowel perforation. Intraoperatively, skip ischemic lesions were observed in the ascending colon close to the hepatic flexure and the rectum without bowel perforation. Although SMA and superior rectal arterial pulsations were present, the marginal perfusion near the two lesions could not be confirmed. The patient was diagnosed with NOMI based on these operative findings and the rapid progression of the symptoms, which are unlike other vascular disorders or necrotizing enterocolitis. The remaining colon, from the transverse to the sigmoid colon, appeared intact. The color of the unaffected intestinal wall was restored, which suggested intestinal viability (Fig. 2). We performed a distal ileostomy without bowel resection because a second-look laparotomy after 24 to 48 h was considered.

After returning to the ICU, the patient required resuscitation for cardiac arrest, septicemia, and DIC. The scheduled second-look laparotomy was canceled, and intensive care including hemodiafiltration was continued. However, the gastrointestinal symptoms did not progress during the intensive treatment.

**Fig. 3:**
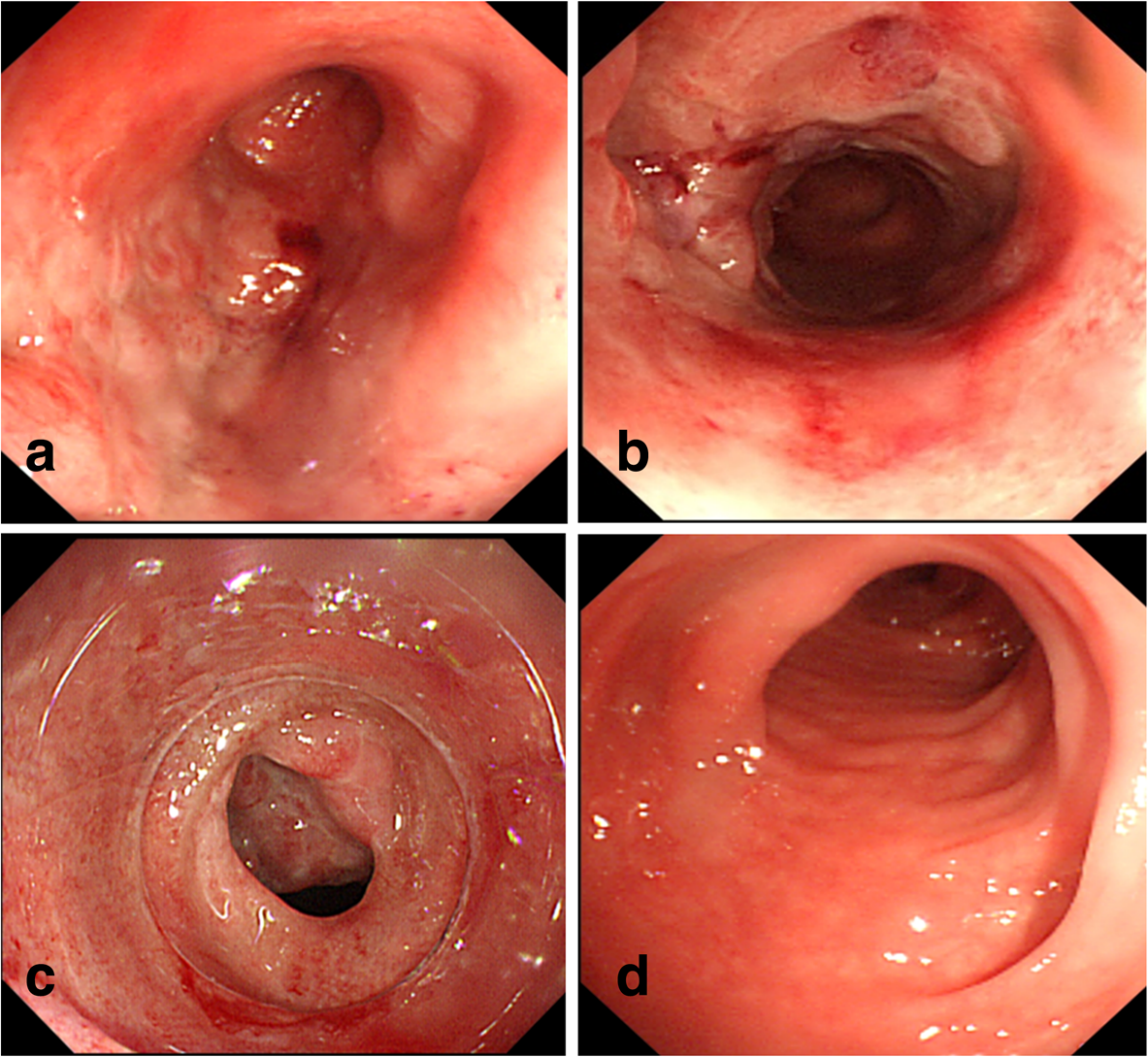
Postoperative colonoscopic findings. On the 60th postoperative day, colonoscopy revealed bowel stenosis with ulceration in the ascending colon (**a**) and the rectum (**b**, **c**). The patency was 5 mm in diameter at both strictures. Colonoscopy showed normal luminal findings through the transverse colon (**d**) to the sigmoid colon

On the 16th and 60th postoperative days, colonoscopies were carried out, and they revealed rectal and ascending colon stenosis with ulceration (Fig. 3). The patency was 5 mm in diameter at both strictures. However, normal findings in the transverse colon to the sigmoid colon were observed by colonoscopy.

**Fig. 4:**
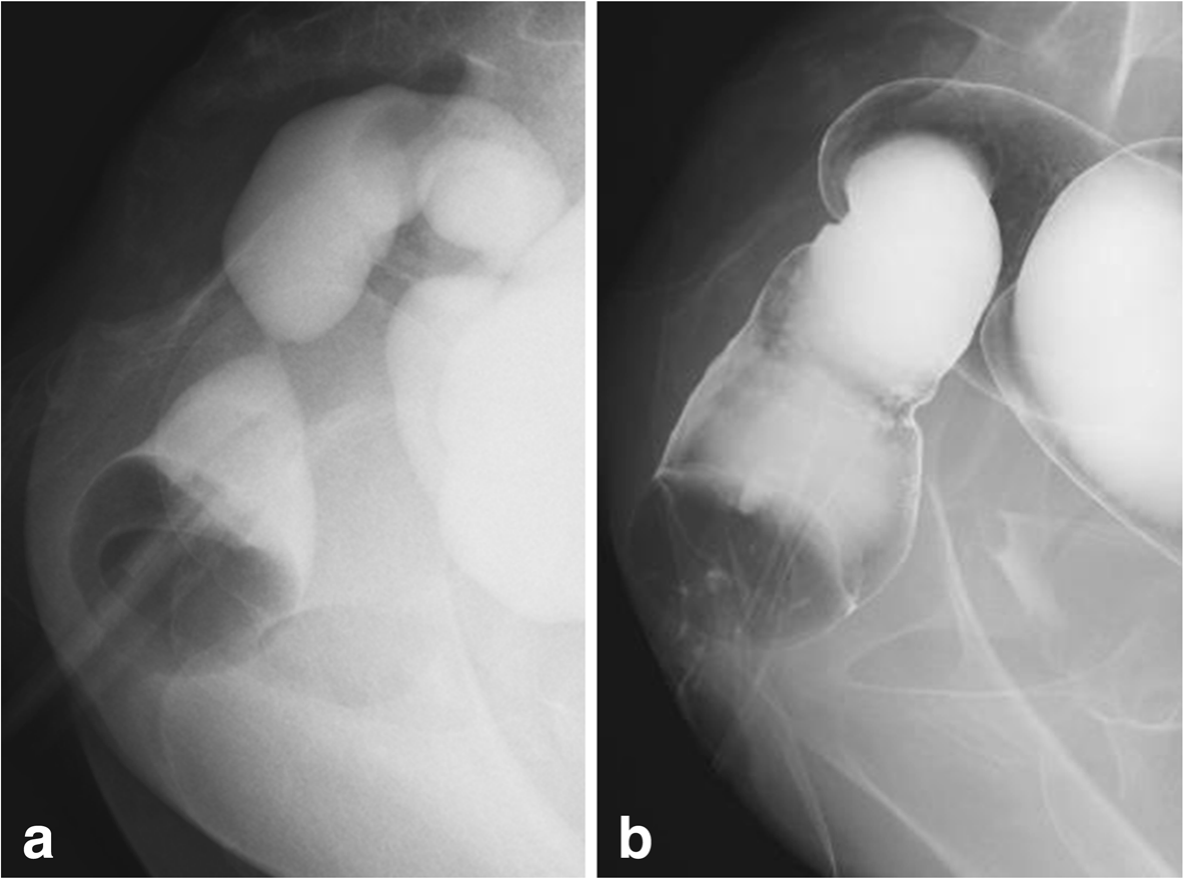
Postoperative lower gastrointestinal (GI) series. Lower GI series by gastrografin at 7 months after laparotomy demonstrated rectal stenotic lesions (**a**). However, successful evacuation of the contrast media was confirmed despite the present stenosis. The stenotic lesion was resolved and defecation has been maintained after stoma closure (**b**)

A lower gastrointestinal series by gastrografin contrast radiography also demonstrated the patency of the two lesions after laparotomy (Fig. 4a). Based on successful evacuation of the contrast media and intact mucosal findings around the mild stricture, we scheduled ileal stoma closure. For 1 month prior to the closure, approximately 100 ml of bowel contents that had collected in the ostomy pouch were injected into the anal side of the ileostomy to induce efficient bowel movement. We confirmed the continuous expulsion of feces from the anus and the improvement of transient bowel strictures; therefore, the ileal stoma was closed 14 months after the previous laparotomy.

Currently, the patient’s confusional state has prolonged, and he has received enriched liquid nutrition via gastrostomy. The two stenotic lesions are completely resolved, and defecation has been maintained after stoma closure (Fig. 4b).

## Discussion

While most NOMI patients are elderly, this phenomenon occurred in a pediatric patient in this present case. In addition, NOMI occurred concurrently in the ascending colon and rectum, to which mesenteric blood flow was supplied via the SMA and IMA, respectively. Finally, we treated delayed intestinal stenosis without resection of the strictures.

We confirm that there are eight reports of NOMI in pediatric cases published in English, referring to the citation of PubMed and a previous report by Jeican, et al. [5]. The representative underlying diseases included familial dysautonomia [8], Addison’s disease [9], situs inversus abdominus [10], burns [11], and chemotherapy administration [12]. However, there is little precise information available in the literature.

The pathophysiology of NOMI is based on vasospasm, which may be related to the “diving reflex,” associated with a homeostatic mechanism that sustains peripheral splanchnic circulation and maintains circulation for vital organs, such as the brain [13]. The possibility of developing intestinal ischemia depends on the extent of systemic perfusion, the affected collateral circulation of mesenteric vessels, and the duration of the ischemic insult [14, 15]. Sensory innervation and a complementary mechanism, such as the nitric oxide-dependent modulation of vascular function in mesenteric arteries, decrease with advanced age [16]. Our patient might have had a physical condition similar to that of elderly adults. Furthermore, we speculated that the underlying disease of limbic encephalitis could be a predisposing factor for NOMI because it affects the structure of the limbic system, which regulates the autonomic nervous system and cardiovascular function. Although children are presumed to have better intestinal blood flow regulation in the mesenteric arteries, we postulate that NOMI may develop in children if the patients have severe underlying disease.

In this case, NOMI presented as skip lesions in the ascending colon and rectum, interposed by the intact transverse and sigmoid colon. The right colic arterial branch of the SMA supplies blood flow to the ascending colon and the rectal branch of the IMA supplies flow to the rectum. There are only a few case reports of NOMI localized in the colon [6, 7]; however, there is a report that indicates that NOMI occurs in the colon in many patients [17]. Thus, it is not fully understood whether NOMI in the colon is common or not. Notably, a previous report stated that the right-side colon appears to be particularly sensitive to NOMI because this site frequently lacks a well-developed and consistent marginal collateral vascular network [4]. There have been no previous reports on NOMI of the rectum. We speculate that the root of the rectal artery originating from the inferior mesenteric artery was maintained while the peripheral branch adjacent to the rectum was selectively affected, even if the rectum had a well-developed collateral vessel network in our case.

We avoided a second-look laparotomy due to the patient’s extremely poor general condition and did not perform a resection of the strictures in the ascending colon and the rectum after the first laparotomy. Severe ischemia mainly causes transmural necrosis followed by fibrosis, even if the intestinal blood flow is restored [18–20]. The main therapeutic treatment of NOMI is resection of the necrotic intestine. If delayed strictures occur, they should be also resected with a multistep surgery. However, we propose that the strictures may be relieved by the intestinal contents passing through the stenotic lesion nearly as efficiently as a bougie if the gastrografin enema demonstrates patency of the lumen. This conservative treatment should be tailored to the patient’s condition, as seen in our case, and continuous observation is necessary to assess the recurrence of delayed stenosis.

## Conclusion

In conclusion, NOMI can present in childhood during encephalitis treatment and can be segmentally localized in the ascending colon and the rectum. Although NOMI is most often seen in elderly patients, we should also consider the possibility of NOMI when pediatric patients with severe illness manifest abdominal symptoms. Additional reports will be needed to reveal whether NOMI may present in children more frequently than previously considered and to elucidate whether mesenteric blood flow in the SMA and IMA can be reduced simultaneously.

## Abbreviations

IMA: Inferior mesenteric arterial
NOMI: Non-occlusive mesenteric ischemia
SMA: Superior mesenteric artery

## References

[1] Ende N (1958). Infarction of the bowel in cardiac failure. N Engl J Med.

[2] Oldenburg WA, Lau LL, Rodenberg TJ, Edmonds HJ, Burger CD (2004). Acute mesenteric ischemia: a clinical review. Arch Intern Med.

[3] Yukaya T, Saeki H, Taketani K, Ando K, Ida S, Kimura Y (2014). Clinical outcomes and prognostic factors after surgery for non-occlusive mesenteric ischemia: a multicenter study. J Gastrointest Surg.

[4] Landreneau RJ, Fry WJ (1990). The right colon as a target organ of nonocclusive mesenteric ischemia. Case report and review of the literature. Arch Surg.

[5] Jeican II, Ichim G, Gheban D (2016). Intestinal ischemia in neonates and children. Clujul Med.

[6] Mitsuyoshi A, Obama K, Shinkura N, Ito T, Zaima M (2007). Survival in nonocclusive mesenteric ischemia: early diagnosis by multidetector row computed tomography and early treatment with continuous intravenous high-dose prostaglandin E(1). Ann Surg.

[7] Murono K, Ishihara S, Kawai K, Kaneko M, Sasaki K, Yasuda K (2017). Non-occlusive mesenteric ischemia localized in the transverse colon: a case report. Surg Case Rep.

[8] Kornecki A, Shemie SD, Daneman A, Ein S (1999). Nonocclusive small bowel infarction in familial dysautonomia syndrome. J Pediatr Surg.

[9] Roldan-Martin MB, Rodriguez-Ogando A, Sanchez-Galindo AC, Parente-Hernandez A, Luengo-Herrero V, Sanchez-Sanchez C (2013). Rare presentation of shock and acute mesenteric ischemia secondary to acute adrenal insufficiency in an 11-year-old male. J Paediatr Child Health.

[10] Mirza B, Ahmad S, Iqbal S, Talat N, Saleem M (2014). Intraoperative acute mesenteric ischemia: a hard nut to crack. J Indian Assoc Pediatr Surg.

[11] Groger A, Bozkurt A, Franke E, Hornchen H, Steinau G, Piatkowski A (2005). Ischaemic necrosis of small and large intestine in a 2-year-old child with 20% partial thickness burns: a case report. Burns.

[12] Hirabayashi K, Takatsuki M, Motobayashi M, Kurata T, Saito S, Shigemura T (2017). Nonocclusive mesenteric ischemia after chemotherapy in an adolescent patient with a history of three allogeneic hematopoietic stem cell transplantations for acute lymphoblastic leukemia. Pediatr Neonatol.

[13] Gooden BA (1993). The evolution of asphyxial defense. Integr Physiol Behav Sci.

[14] Boley SJ, Agrawal GP, Warren AR, Veith FJ, Levowitz BS, Treiber W, Dougherty J (1969). Pathophysiologic effects of bowel distention on intestinal blood flow. Am J Surg.

[15] McKinsey JF, Gewertz BL (1997). Acute mesenteric ischemia. Surg Clin North Am.

[16] Guzik TJ, Touyz RM (2017). Oxidative stress, inflammation, and vascular aging in hypertension. Hypertension.

[17] Cocorullo G, Mirabella A, Falco N, Fontana T, Tutino R, Licari L (2017). An investigation of bedside laparoscopy in the ICU for cases of non-occlusive mesenteric ischemia. World J Emerg Surg.

[18] Glotzer DJ, Villegas AH, Anekamaya S, Shaw RS (1962). Healing of the intestine in experimental bowel infarction. Ann Surg.

[19] Mitsudo S, Brandt LJ (1992). Pathology of intestinal ischemia. Surg Clin North Am.

[20] Maezawa S, Fujita M, Sato T, Kushimoto S (2015). Delayed intestinal stricture following non-resectional treatment for non-occlusive mesenteric ischemia associated with hepatic portal venous gas: a case report. BMC Surg.

